# Temperature mapping of non-photochemical quenching in *Chlorella vulgaris*

**DOI:** 10.1007/s11120-022-00981-0

**Published:** 2022-11-22

**Authors:** Andrei Herdean, Christopher Hall, David J. Hughes, Unnikrishnan Kuzhiumparambil, Bernardo Campos Diocaretz, Peter J. Ralph

**Affiliations:** Climate Change Cluster, University of Technology Sydney, Ultimo, NSW 2007 Australia

**Keywords:** Phenoplate, Temperature, NPQ

## Abstract

Light intensity and temperature independently impact all parts of the photosynthetic machinery in plants and algae. Yet to date, the vast majority of pulse amplitude modulated (PAM) chlorophyll *a* fluorescence measurements have been performed at well-defined light intensities, but rarely at well-defined temperatures. In this work, we show that PAM measurements performed at various temperatures produce vastly different results in the chlorophyte *Chlorella vulgaris*. Using a recently developed Phenoplate technique to map quantum yield of Photosystem II (Y(II)) and non-photochemical quenching (NPQ) as a function of temperature, we show that the fast-relaxing NPQ follows an inverse normal distribution with respect to temperature and appears insensitive to previous temperature acclimation. The slow-relaxing or residual NPQ after 5 minutes of dark recovery follows a normal distribution similar to Y(II) but with a peak in the higher temperature range. Surprisingly, higher slow- and fast-relaxing NPQ values were observed in high-light relative to low-light acclimated cultures. Y(II) values peaked at the adaptation temperature regardless of temperature or light acclimation. Our novel findings show the complete temperature working spectrum of Y(II) and how excess energy quenching is managed across a wide range of temperatures in the model microalgal species *C. vulgaris*. Finally, we draw attention to the fact that the effect of the temperature component in PAM measurements has been wildly underestimated, and results from experiments at room temperature can be misleading.

**Supplementary Information:**

The online version contains supplementary material available at 10.1007/s11120-022-00981-0.

## Introduction

The challenge of dissipating excess absorbed light energy is something that all photosynthetic organisms must overcome in natural environments (Ruban et al. 2012). The processes that such organisms use to achieve this are categorised under the umbrella term of photoprotective mechanisms. The most common way of evaluating and quantifying photoprotective properties of an organism is through chlorophyll *a* fluorescence measurements. For example, non-photochemical quenching (NPQ) is a parameter derived from such measurements that is used to characterise photoprotective capacity (Müller et al. 2001; Baker 2008). NPQ can be deconvoluted to describe several biological mechanisms occurring over different timeframes that work to protect the organism from damage caused by excess light. In the microalga *Chlorella vulgaris*, two such mechanisms have been identified and described: energy-dependent quenching (qE) and photoinhibition (qI) (Quaas et al. 2015; Finazzi and Minagawa 2014). State transition (qT) represents an additional photoprotective mechanism which has been reported to have a negligible contribution in high-light (Quaas et al. 2015) but significant in low-light (Bonaventura and Myers 1969). In the microalgae *Chlamydomonas reinhardtii*, it has further been shown that an additional zeaxanthin component (qZ) of NPQ can be observed; however, it overlaps with the other photoprotective mechanisms (Erickson et al. 2015). In the dinoflagellate *Symbiodinium sp.* which is commonly found in endosymbiosis with corals a spill-over type of quenching mechanisms has been documented where excitation energy is transferred from Photosystem II (PS(II)) directly to Photosystem I (PS(I)) during a process called super-quenching (Slavov et al. 2016). In higher plants, specifically in the model plant *Arabidopsis thaliana*, two more components of NPQ have been identified (i) photo avoidance (qM) (Cazzaniga et al. 2013) and (ii) sustained photoprotective energy dissipation (qH) (Brooks et al. 2013; Malnoë et al. 2017). Collectively, these processes work in concert to allow plants and microalgae to grow in conditions of fluctuating light intensity and spectrum found in the natural environment. Several of these NPQ components have been demonstrated to work together and to be tightly connected: qE requires zeaxanthin to fully function in *Nannochloropsis oceanica* (Short et al. 2022), in *Chlamydomonas reinhardtii* it needs both zeaxanthin and loroxanthin to reach maximum values (Niyogi et al. 1997), in plant leaves with pre-established zeaxanthin content qE is accelerated (Demmig-Adams et al. 1989), and zeaxanthin has also been associated with conditions of photoinhibition (qI) (Adams III et al. 2006; Adams et al. 1995).

In the past, significant research effort has been dedicated towards describing the relationship of NPQ with light, but little investigation has been dedicated to addressing the impact of fluctuating temperatures that often accompany changes in light intensity (Salleh and McMinn 2011; Behrendt et al. 2020; Slavov et al. 2016). A general complication in determining the thermal properties of complex photosynthetic mechanisms is the global impact that temperature has upon the entire metabolism of the cell, not just photosynthesis. Therefore, to precisely determine the temperature-associated kinetics of photosynthesis, the organism needs to be brought rapidly, and held briefly, to various temperatures. Additionally, it is crucial that the new temperatures are precisely controlled and maintained constant during subsequent photosynthetic measurements.

In the present study, we aimed to describe the temperature properties of photoprotection and quantum yield of PS(II). We did this by performing a rapid temperature change during NPQ induction and relaxation measurements. Specifically, fast- and slow-relaxing NPQ, NPQ rise in darkness, ± Far-Red light (FR)-responsive NPQ, and quantum yield of Photosystem II (Y(II)) were measured over a temperature range of 10 to 45 °C using the previously described Phenoplate system (Herdean et al. 2022). Our results provide novel insight into the activation and relaxation of different photosynthetic mechanisms in relation to temperature.

## Results

**Fig. 1:**
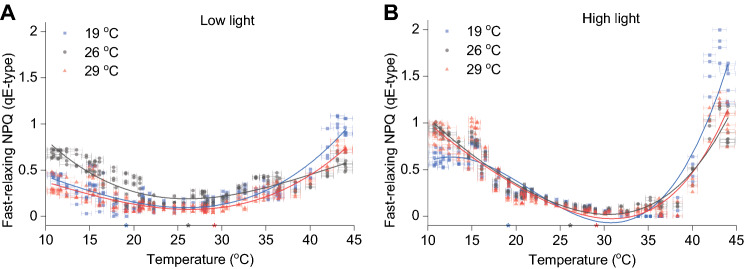
qE-type NPQ temperature mapping. Samples grown in different temperatures (19, 26 and 29 °C) in low-light (**A**) or high-light (**B**) measured using the Phenoplate across multiple temperature gradients. Coloured stars on the temperature axis indicate the growth temperature of the three cultures. Lines indicate fitted data with a 3^rd^ order polynomial function using OriginPro. Data presented are results from four Phenoplate measurements using different temperature gradients, and four biological replicates for each acclimated condition (*n* = 4). The experiment has been replicated after 6 and 12 months and yielded similar results

The amplitude of the fast-relaxing NPQ (qE-type) was quantified at various temperatures using a specially designed protocol described in Materials and Methods (see also Supplementary Figure S1). In all low-light acclimated samples, the qE-type NPQ followed an inverse normal distribution curve reaching maximum values in low and high temperature, < 20 °C and > 30 °C, respectively (Fig. 1A). A similar data distribution was observed in high-light acclimated samples; however, the data were skewed towards higher temperatures (Fig. 1B). This type of NPQ was observed to reach minimum values at ~ 26 °C in low-light and ~ 30 °C in high-light acclimated samples (Table 1). Little to no differences were observed in the temperature response kinetics between samples acclimated to different temperatures, with the exception of the 19 °C high-light samples which showed reduced quenching in low temperature and higher quenching in high temperature relative to the 26 °C and 29 °C high-light acclimated samples (Fig. 1B).

**Table 1:** Data fitting values of interest

	Peak/dip temperature (^o^C)			Amplitude of peak/dip		
	19 °C	26 °C	29 °C	19 °C	26 °C	29 °C
qE-type NPQ (LL)	26.1 ± 1.5	25.9 ± 1.2	26.0 ± 1.4	0.190	0.094	0.073
qE-type NPQ (HL)	30.8 ± 0.1	30.4 ± 0.5	30.9 ± 0.3	− 0.071	− 0.019	− 0.027
qT_2_-type NPQ (LL^+FR^)	34.1 ± 0.1	34.5 ± 0.2	35.3 ± 0.1	0.353	0.238	0.384
qT_2_-type NPQ (HL^+FR^)	34.6 ± 0.1	35.2 ± 0.1	35.6 ± 0.1	0.648	0.325	0.590
qT_2_-type NPQ (LL^−FR^)	34.1 ± 0.1	34.6 ± 0.6	34.5 ± 0.1	0.510	0.355	0.625
qT_2_-type NPQ (HL^−FR^)	34.6 ± 0.1	35.5 ± 0.1	36.2 ± 0.1	0.722	0.443	0.738
slow-relaxing NPQ (LL)	40.5 ± 0.2	40.9 ± 0.4	41.1 ± 0.2	1.347	0.772	1.092
slow-relaxing NPQ (HL)	39.9 ± 0.1	40.5 ± 0.2	39.8 ± 0.1	2.747	1.598	1.910
Y(II) (LL)	28.5 ± 0.1	28.6 ± 0.5	30.4 ± 0.2	0.292	0.260	0.333
Y(II) (HL)	30.9 ± 0.5	30.1 ± 0.3	30.9 ± 0.5	0.368	0.320	0.415

**Fig. 2:**
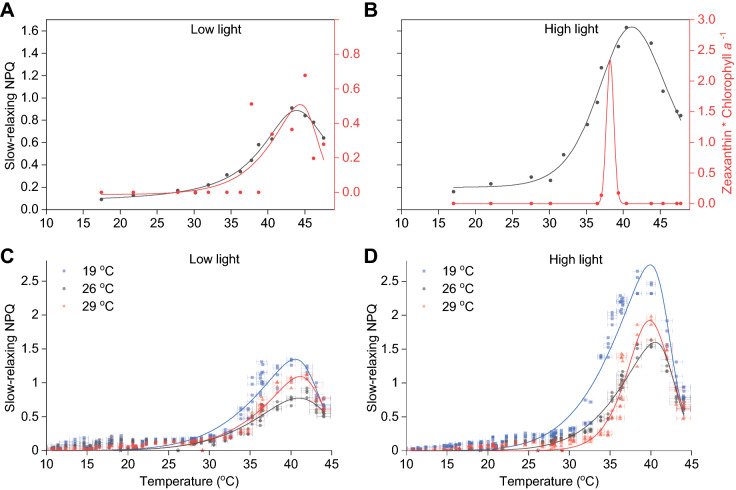
Slow-relaxing NPQ and zeaxanthin production temperature mapping. Samples acclimated to 26 °C were measured using the Phenoplate and analysed subsequently using HPLC in order to quantify their zeaxanthin content (**A**, **B**). Slow-relaxing NPQ and zeaxanthin are shown for selected samples across the entire tested temperature range for low (**A**) and high (**B**) light acclimated cultures. Samples grown in different temperatures (19, 26 and 29 °C) in low-light (**C**) or high-light (**D**) measured using the Phenoplate across multiple temperature gradients. Coloured stars on the temperature axis indicate the growth temperature of the three cultures. Lines indicate fitted data with a Biphasic Hill Growth function using OriginPro. Data presented are results from four Phenoplate measurements using different temperature gradients, and four biological replicates for each acclimated condition (*n* = 4)

**Fig. 3:**
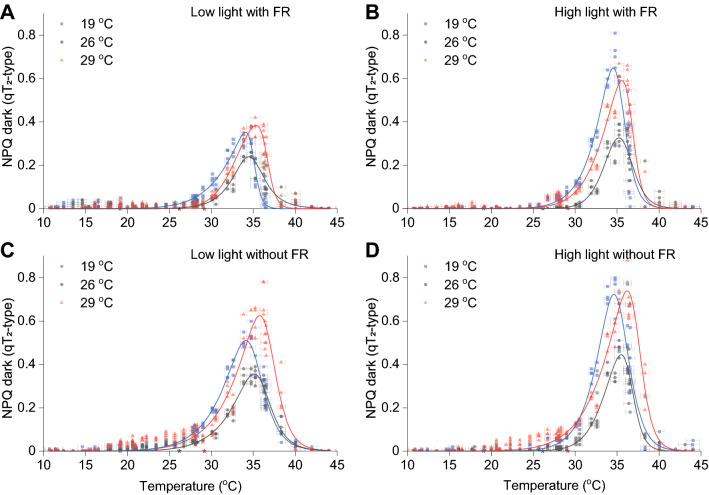
qT_2_-type NPQ temperature mapping. State transition to state 2 of samples acclimated to different temperatures (19, 26 and 29 °C) in low-light (**A**) or high-light (**B**) with far-red turned on during dark recovery. qT_2_-type NPQ of samples acclimated to different temperatures (19, 26 and 29 °C) in low-light (**C**) or high-light (**D**) with far-red turned off during dark recovery measurements. Coloured stars on the temperature axis indicate the growth temperature of the three cultures. Lines indicate fitted data with a Biphasic Hill Growth function using OriginPro. Data presented are results from four Phenoplate measurements using different temperature gradients, and four biological replicates for each acclimated condition (*n* = 4)

**Fig. 4:**
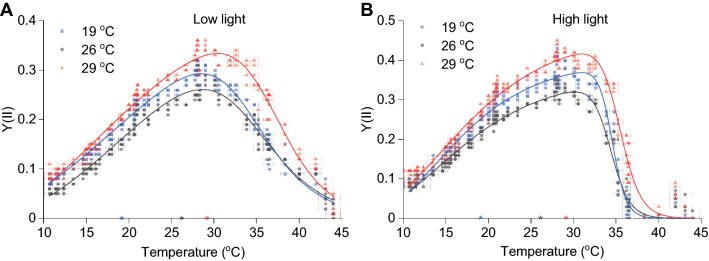
Y(II) temperature mapping. Data points represent Y(II) values collected after five minutes of illumination with high-light (500 μmol photons m^−2^ s^−1^) at the indicated temperatures on the vertical axis. Coloured stars on the temperature axis indicate the growth temperature of the three cultures. Lines indicate fitted data with a Biphasic Hill Growth function using OriginPro. Data presented are results from four Phenoplate measurements using different temperature gradients, and four biological replicates for each acclimated condition (*n* = 4)

Extracted values from the peaks or dips of the data fittings from Figs. 1–4. Each column corresponds to samples acclimated to different temperatures (19, 26 and 29 °C). The first three columns indicate the position of the peak or dip on the temperature (horizontal) axis, and the last three columns indicate the maximum or minimum value reached on the vertical axis. Each row corresponds to different parameters and different light acclimation (LL low-light; HL high-light)

“^+FR^” indicate values from samples that were exposed to far-red (FR) light during dark recovery

“^−FR^” indicate values from sample that were not exposed to far-red light during dark recovery

Maximum fluorescence (F_m_^'^) in the dark phase of our experiments did not fully relax within the allocated five minutes, suggesting that slow-relaxing NPQ components were still present. The illumination time was sufficient for the xanthophyll cycle to accumulate zeaxanthin (Girolomoni et al. 2020) but also sufficient for a certain degree of photodamage to the D1 subunit of PS(II) (Aro et al. 1993). Therefore, we assumed that the quenched fluorescence that did not relax after 5 minutes likely represented a combination zeaxanthin-induced quenching, photoinhibition and other factors. We further investigated which of the components were more dominant in our data by quantifying the relative amount of zeaxanthin using high-performance liquid chromatography (HPLC). Surprisingly, the slow-relaxing NPQ component followed an almost identical distribution with zeaxanthin content in low-light acclimated samples but not in high-light acclimated cultures (Fig. 2A, B).

The slow-relaxing NPQ peaked at 40 °C irrespective of culture acclimation temperature or light (Fig. 2C, D). This may be indicative of the working temperature range of enzymes involved in the xanthophyll cycle and/or D1 damage and degradation. The highest slow-relaxing NPQ was recorded in low temperature acclimated samples when exposed to high temperatures (Fig. 2D). Data distribution and peak position on the temperature axis were very similar for all samples (Table 1). NPQ peaked at ~ 40 °C and was followed by a decline in the 40 to 45 °C range. The lowest slow-relaxing NPQ values were recorded at low temperatures between 10 and 20 °C (Fig. 2C, D).

In the data extracted from our experiments, we observed two types of fluorescence quenching during dark acclimation: (i) a far-red light sensitive change in NPQ which was defined as the difference in NPQ during dark recovery in the presence and absence of far-red (± FR) light, similar to state transition to state 1 (qT_1_) (Lunde et al. 2000), (ii) and a steady increase of NPQ in the absence of light irrespective of FR light, similar to state transition to state 2 (qT_2_). The FR-responsive NPQ was detected mostly in high-light acclimated samples and did not follow any discernible patterns (Supplementary Fig. 2A, B). The use of far-red light during dark acclimation had no secondary effect on the other NPQ components (Supplementary Fig. 3). The steady increase of NPQ in dark was present in all samples regardless of temperature, light acclimation, or the presence/absence of far-red light (Fig. 3A–D). This type of NPQ was active only between 30 and 40 °C, with a clear peak at ~ 35 °C. Sample acclimation appeared to only influence the amplitude of this type of NPQ response.

In low-light acclimated cultures, measured Y(II) values for 19 °C and 26 °C acclimated cultures were remarkably similar (Fig. 4A), with maximum Y(II) being reached at ~ 28 °C compared to at ~ 30 °C in all other acclimated cultures (Fig. 4A, B, Table 1). Interestingly, high-light acclimated cultures experienced an abrupt drop in Y(II) at temperatures of 35 °C and over (Fig. 4B). Towards either end of the temperature spectrum (i.e. cold or warm), Y(II) values were generally lower; however, towards the warm end of the spectrum, low Y(II) values were also accompanied by increased slow-relaxing NPQ and zeaxanthin (Figs. 2, 4). This follows a thermal performance curve type of rate dependence with increasing temperature.

**Fig. 5:**
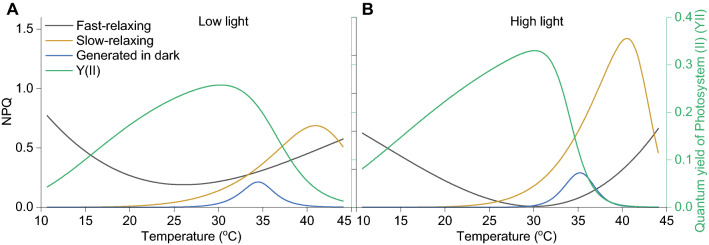
Global view of temperature response kinetics of photoprotection and PS(II) quantum yield. Data fittings only of recorded data from *C. vulgaris* cultures acclimated to 26 °C and low-light (A) or high-light (B). Fitting parameters are as described in legends of preceding figures

Lastly, all NPQ components were compared to one another to visualise the relationship of the entire photoprotective system to temperature (Fig. 5). The data revealed that Y(II) peaked at temperatures where qE-type fast-relaxing NPQ was low, and just before the onset of slow-relaxing NPQ. Fluorescence quenching generated in the dark appeared closely after Y(II) started to decline and was active for only a brief temperature interval. The fast-relaxing NPQ was the only type of fluorescence quenching mechanism detected at low temperatures, with neither slow-relaxing or FR sensitive NPQ, or zeaxanthin observed at temperatures lower than 25 °C. Kinetics of the NPQ that did not relax within the allocated 5 minutes started with a slow rise from ~ 20 °C, followed by a peak at ~ 40 °C, after which it started declining. The qE-type NPQ continued to rise even after the slow-relaxing NPQ started declining at temperatures > 40 °C. Light intensity acclimation affected the amplitude of qE-type and slow-relaxing NPQ, the working temperature distribution of Y(II), and had minimal impact on the NPQ generated in dark (Fig. 5A, B).

## Discussion

In this work, we set out to determine the working temperature range of NPQ in *C. vulgaris*. The fast-relaxing qE-type of NPQ likely relies on the presence of carotenoid-type pigments such as zeaxanthin (Short et al. 2022) and a proton gradient across the thylakoid membrane. It is therefore directly impacted by the xanthophyll cycle, the functioning of the oxygen evolving complex (OEC), cytochrome *b*_6_*f* and ATP synthase (Davis et al. 2017). This is of course a simplified model which does not take into account cyclic electron flow, or proton gradient consumers other than ATP synthase (Munekage et al. 2002; Armbruster et al. 2014).

When the balance between proton accumulation and consumption in the thylakoid lumen is disrupted, the ∆pH across the thylakoid membrane is altered which in turn triggers an immediate response of NPQ (Ruban 2016). As such, the qE-type NPQ temperature mapping could reflect the thermal properties of either proton gradient generators, consumers, or effectors (or indeed a combination of all). Furthermore, the qE-type NPQ is dependent on availability of zeaxanthin and lutein (Niyogi et al. 2001), which are also affected by temperature. We assumed that all components of the photosynthetic machinery are intrinsically connected to such an extent that it is impractical to attempt a deconvolution of thermal properties into effectors or elicitors. Therefore, we considered our observations to reflect light and thermal properties of a complete, multi-component functional photosynthetic machinery.

In our experimental setup, we could separate the temperature response of fast-relaxing NPQ into two parts: NPQ in low temperature, and NPQ in high temperature. In low temperature, all NPQ relaxed within the 5 minutes of dark acclimation and was negatively correlated with Y(II). The fast-relaxing NPQ response in high temperature was similar, but associated with other components such as slow-relaxing, FR-responsive and dark generated NPQ, and as in low temperature negatively correlated with Y(II). Furthermore, non-photochemical quenching at elevated temperatures appeared to increasingly rely on regulated processes rather than unregulated energy loss mechanisms (Supplementary Fig. 4). The negative correlation with Y(II) was expected and is consistent with an energy sink model of photosynthesis, whereby excitation energy is quenched if it cannot be used by PS(II). These data suggest that in studies where the sole effect of qE-type of NPQ is to be observed, measurements performed at reduced temperatures could prove extremely insightful.

Previously, Tikhonov and Vershubskii published a comprehensive model using experimental data and modelling where the temperature dependency of ATP production was shown to follow a bell curve distribution across a temperature axis (Tikhonov and Vershubskii 2020). This temperature dependence was modelled on the light reactions only, with the primary contributors being the oxidation rate of cytochrome *b*_6_*f*, temperature inactivation of PS(II), and changes in passive proton permeability of the thylakoid membrane. Unlike the Tikhonov and Vershubskii, our observed Y(II) temperature response curve did not fit a bell curve, but rather approximated an asymmetric normal distribution. However, qE-type NPQ followed an inverted normal distribution which is consistent with their model, where high qE-type NPQ would correspond to low ATP production and vice versa. Taken together, this suggests that the bell curve model of ATP production partially explains the impact of temperature on photosynthetic efficiency, and that there are other important factors not captured by their model.

It is interesting and unexpected to note that while temperature acclimation had little to no effect on the qE-type NPQ thermal response, light acclimation clearly did. This can be interpreted as qE being the result of a sum of molecular mechanisms that have intrinsic thermal properties and inducible light properties. The same statement can be made in respect to the other types of NPQ detected, as all these photoprotective mechanisms appear to have a preferred temperature where they were activated (Fig. 5).

Far-red-responsive qT_1_-type NPQ occurred to a small extent and sporadically at elevated temperatures (Supplementary Fig. 2AB). This was expected since in conditions of high-light the light-harvesting antennas are known to be connected preferentially to PS(II), therefore having the light-harvesting system already in the state 1 condition (Allorent et al. 2013). Nonetheless, the observed far-red-responsive NPQ was predominantly detected in 19 °C and 29 °C high-light acclimated cultures which suggests that this photoprotective resource was not fully required in the other tested condition, or the light-harvesting complex II (LHCII) pool was insufficiently phosphorylated during the illumination phase.

During dark recovery, we observed two fluorescence kinetic phases that have been previously documented in *Chlamydomonas reinhardtii*: phase IV—a fast recovery of F_m_′ corresponding to relaxation of the qE quenching mechanism, and phase V—a subsequent quenching of F_m_′ in darkness assigned to transition from state 1 to state 2 (qT_2_) (Allorent et al. 2013) (Supplementary Fig. 5). As expected, the extent of state 1 to state 2 transition in darkness was impacted by far-red light which can be seen in the reduced amplitude of the NPQ generated in dark in measurements with far-red light relative to measurements in the absence of far-red light (Fig. 3). Phase IV was observed in all tested conditions which is in supports of our assessment of fast-relaxing NPQ as a qE-type (Fig. 1) and the presence of phase I in light which corresponds to induction of qE. However, phase V was absent in low and high temperatures which supports that dark generated NPQ is a qT_2_-type of quenching (Fig. 3). Transition from state 1 to state 2—which occurs shortly after starting sample illumination and is observable in the phase II of the fluorescence induction kinetics—was missing in all low temperature measurements. This suggests that at low temperature the photosynthetic light-harvesting system remains locked in state 1, a phenomenon that can be explained by inability of kinases to phosphorylate LHCII (Depège et al. 2003), possibly due to transient inactivation at low temperature. While the relationship between such kinases and temperature has not been studied in plants or algae, it has been studied in the yeast *Saccharomyces cerevisiae* where kinase activity follows a normal distribution with inactivation occurring at low or elevated temperatures (Chauhan et al. 2019).

Dark generated NPQ appeared over a 10 °C temperature range when Y(II) was declining, and all other NPQ types and zeaxanthin were increasing. Our data suggest that the increase of NPQ in darkness is triggered by a process that is linked to pre-illumination with high-light in the temperature range where qE-type of quenching is not fully active, and slow-relaxing type of NPQ is increasingly dominant.

High temperatures are known to inhibit the photosystem II repair cycle (Allakhverdiev et al. 2008), and thus the temperature mapping of the slow-relaxing NPQ which peaked at elevated temperatures was consistent with our expectations that it reflects to some extent photodamage. However, unexpectedly this type of NPQ started decreasing at temperatures above 40 °C even when the fraction of closed reaction centres continue to increase (Supplementary Fig. 6). This result may reflect multiple overlapping processes such as limitation to photosystem II repair (Wu et al. 2017), damage to the OEC (Tóth et al. 2005), PS(II) loss of function due to D1 degradation and accumulation of zeaxanthin (Adams III et al. 2006). Future work could address each of these possibilities by re-designing the experimental protocol, to allow quantification of D1 protein content and an in-depth quantification of the xanthophyll pigments.

Temperature mapping of the PS(II) quantum yield (Y(II)) provided a very different insight into the dynamics of photosynthesis. We expected that 60 days of temperature acclimation (Raven and Geider 2003) would shift the optimum of Y(II) to a new temperature, yet this was not the case. Even after 12 months of sample acclimation, the results were unchanged (data not shown). Acclimation to different light intensities also did not change the temperature optimum, but did alter Y(II) behaviour at elevated temperatures. This latter observation could possibly be the result of an altered accessory pigment composition, not directly from chlorophyll a:b which was found to be similar in most samples (Supplementary Table 1). While the optimum temperature for Y(II) was not altered, we did observe significant changes in F_v_/F_m_ values. The highest measured F_v_/F_m_ was in low temperature acclimated cultures, while the lowest F_v_/F_m_ value was observed at 26 °C under high-light (Supplementary Fig. 7).

## Conclusions

In the present study, we performed a set of experiments that demonstrate that the thermal properties of photosynthesis and photoprotection are not temperature inducible, at least not in *C. vulgaris*, and not over 60 days. We show that NPQ can be deconvoluted into components that relax fast or slowly, are responsive to FR or are generated during dark acclimation and can be temperature mapped. Our study offers detailed insight into the temperature dependency of photoprotection and photosynthetic efficiency across a broad temperature range. The data show that *C. vulgaris* has a single fast acting photoprotective response in conditions of reduced temperature and a complex multi-component response in elevated temperatures. This approach of performing PAM measurements can be used in conjunction with loss-of-function mutants to further investigate how the kinetics of fast and slow-relaxing NPQ are modulated at different temperatures by proteins that are known to have a direct role in NPQ, such as PGRL1 (Petroutsos et al. 2009), PGR5 (Johnson et al. 2014), PsbS (Li et al. 2000), Stt7 (Depège et al. 2003), LHCSR (Peers et al. 2009), VDE and the xanthophyll cycle (Girolomoni et al. 2020). Furthermore, the dynamic changes in thylakoid membrane ultrastructure in response to temperature and the impact of these changes on the slow-relaxing NPQ could be studied in a novel way using our Phenoplate system.

## Materials and methods

### Algae history and growth conditions

*Chlorella vulgaris* strain CS-41 was sourced from the Commonwealth Scientific and Industrial Research Organisation (CSIRO). Prior to the experiments described in this work, the algae was maintained in the Climate Change Cluster (C3) culture collection for at least 5 years in 12 h dark and 12 h light at 20 ± 2 μmol photons m^−2^ s^−1^ white fluorescent light, at 21 ± 0.5 °C day and 19.5 ± 0.5 °C night temperature. CSIRO documents indicate that the organism has been kept in their collection from as early as 2010 and the algae is an exact genetic match with CCAP 211/11 s from the Culture Collection of Algae & Protozoa from the Scottish Association for Marine Science, Oban UK, and SAG 211-11 s from the Culture Collection of Algae at the University of Göttingen, Germany. At C3, the cultures were maintained in MLA media (Algaboost, AusAqua Pty, LTD Wallaroo, SA, Australia) (Bolch and Blackburn 1996) prepared in natural seawater in 70-mL Corning cell culture flasks (item number 353108, Corning Incorporated, NY, USA) each filled with 30 mL culture media. Flasks were kept horizontally in three temperature-controlled rooms under broad spectrum white light provided by either light emitting diodes (LED) or fluorescent tubes. In each condition, four flasks were covered with two layers of 0.3 neutral density filter (#209 LEE Filters, Andover, Hampshire, UK), while four more flasks were left uncovered. Light intensity was measured at 120 ± 5 μmol photons m^−2^ s^−1^ for uncovered cultures referred throughout the text as high-light, and 30 ± 5 μmol photons m^−2^ s^−1^ for neutral density filter-covered cultures referred throughout the text as low-light. Day night cycle was set to 12 h light/12 h dark, starting in all rooms at the same time of the day. Cultures were acclimated to three different temperatures in three temperature-controlled rooms with the following profiles: (1) 18.25 ± 0.25 night and 19 ± 0.25 °C day temperature, samples acclimated in this temperature referred throughout the text as 19 °C acclimated; these cultures were grown using an LED light source; (2) 23.75 ± 0.25 night and 26 ± 0.5 °C day temperature, samples acclimated in this temperature referred throughout the text as 26 °C acclimated; these cultures were grown using an LED light source (3) 22.75 ± 0.25 night and 29 ± 1 °C day temperature, samples acclimated in this temperature referred throughout the text as 29 °C acclimated; these cultures were grown using fluorescent tube as light source. Cultures were maintained in semi-continuous batch culture in each condition for at least 60 days, and approximately, every tenth day, the cultures were diluted by 90% using fresh MLA media.

### Pre-measurement sample preparation

At least 12 h before commencement of measurements, algae culture density was adjusted for all cultures to an optical density of 0.3 at 680 nm (OD 680), as measured with a plate reader (Spark, Tecan Schweiz AG, Männedorf, Switzerland). This was done by 5 minutes centrifugation (600 g) of an appropriate volume of microalgal culture using a Multifuge X Pro Series centrifuge (Thermo Fisher Scientific Inc., Waltham, MA, USA) with a TX-1000 rotor (Nowicka 2020). Supernatant was then carefully removed down to 6 mL, on top of which we added 24 mL of fresh MLA media. The resulting culture suspensions were all at OD 680 of 0.3 in 80% fresh MLA media.

Optically adjusted samples were carefully loaded into a 96-well plate in each of the temperature-controlled rooms in order to avoid unnecessary temperature and light fluctuations. The prepared 96-well plate was then moved to the thermocycler which was set to the growth temperature of the samples. The Phenoplate protocol (Herdean 2021) was immediately initiated after sample delivery to the thermocycler.

### Pigment analysis

Pigment content was determined by filtering 10 mL of algal culture through a 25 mm glass fibre filter (Advantec, GB-100R, nominal pore size 0.3 μm). Filters were then immediately transferred to 20 mL glass vials, in 7.2 mL of 90% acetone was added to the filter and pigments were subsequently extracted for 96 hours under darkness at 4 °C. Chlorophyll *a* and *b* content were calculated according to Ritchie (2006). Sample absorbance was measured with a plate reader (Spark, Tecan Schweiz AG, Männedorf, Switzerland).

A one-way analysis of variance (ANOVA) was used to test for differences in Chl a:b between treatments, using a Tukey HSD post hoc test when significant differences were detected. Prerequisite assumptions of normality and equal variance were first verified using Shapiro–Wilk’s and Levene’s tests, respectively. Analyses were performed using SPSS software (version 20.0, SPSS Inc, Chicago, Illinois, USA).

Zeaxanthin quantification by HLPC was done on samples acclimated for 12 months at 26 °C. Four 96-well plates (item number 353072, Corning Incorporated, NY, USA) were filled with 100 μl acetone (100%v/v) in each well and chilled at − 80 °C. Next, a Phenoplate experiment was carried out, and immediately after completion of the measurement the 96-well plate containing the samples was transferred on to an OT-2 liquid handling robot (Opentrons, Brooklyn, New York, USA). The robot was programmed to transfer 100 μl of cell culture from each well in to the 96-well plate which contained the 100 μl chilled acetone. The procedure was carried out under low green light and took 2 minutes and 30 seconds for each plate. The new microplate containing sample and acetone was transferred to − 80 °C and stored overnight. Selected samples were transferred to 1.5-ml microcentrifuge tubes (Eppendorf, Hamburg, Germany) on to which 100 µL chilled acetone (Burdick & Jackson, USA) was added, sonicated for 30 seconds (Unisonics Brookvale, Australia) and then vortexed (Bacto Laboratories, Mount Pichard, Australia) for 3 × 30 seconds under cold, dark conditions and then stored overnight at 4 °C. Pigment extracts were collected after centrifuging at 5000 rpm for 5 minutes at 4 °C (Centrifuge model: 5424R; Eppendorf, Hamburg, Germany) and then filtered through a 0.2 µM PTFE 13-mm syringe filter (Filter-Bio, Nantong, China) and stored in − 80 °C until analysis. An Agilent 1290 HPLC system (Agilent Technologies, Santa Clara, USA) equipped with a binary pump with integrated vacuum degasser, thermostatted column compartment modules, Infinity 1290 autosampler and PDA detector was used for the analysis. An Agilent's Zorbax Eclipse XDB C8 HPLC 4.6 mm × 150 mm and guard column was used for analysis using a gradient of TBAA: Methanol mix (30:70) (Solvent A) and Methanol (Solvent B) as follows: 0–22 minutes, from 5 to 95% B; 22–29 minutes, 95% B; 29–31 minutes, 5% B; 31–40 minutes, column equilibration with 5% B. Column temperature was maintained at 55 °C. A complete pigment spectrum from 270 to 700 nm was recorded using PDA detector with 3.4 nm bandwidth. Peak identification was performed using analytical standards of zeaxanthin and chlorophyll *a* (DHI Pigment, Denmark) and comparing the retention time and absorbance spectra.

### Temperature measurements

Algae growth temperature was measured using temperature loggers (DS1921G#F50, iButtonLink, LLC, Whitewater, WI, USA). Three probes were placed in each room next to the culture flasks and data were recorded for 7 days. Additionally, we measured the temperature using a calibrated Thermal Camera model FLIR C2 (Teledyne FLIR, LLC, Wilsonville, OR, USA) with calibration certification traceable to National Standards at the SP Technical Research Institute of Sweden, or to National Institute of Standards and Technology (USA).

To determine the exact temperature of the sample during our fluorescence measurements, we used the same FLIR C2 thermal camera. We fixed the camera at a 20 cm distance from the Gradient Thermal Cycle (5331 MasterCycler Eppendorf SE, Hamburg, Germany) which was programmed to generate the sequence of temperature gradients which were used for the fluorescence measurements. We placed a HSP9601 (Bio-Rad Laboratories, Inc, Hercules, CA, USA) 96-well plate with each well filled with 200 μL of ultrapure water and recorded a thermal image of the plate every 2 s.

Images were processed using a Python script to read the temperature scale in each thermal image and the extract temperature values for the wells from the thermal camera images. The script is available on Github (https://github.com/DrPlantabyte/Temperature-mapping-FLIR-Analysis-Scripts).

In addition, temperature in each microwell was measured using an array of medical precision thermistors (item number MA100BF103BN, Amphenol Advanced Sensors, St. Marys, PA, USA) coupled to an Arduino Uno R3 (item number SF-DEV-11021, Little Bird Electronics, Hornsby, NSW, Australia). Circuit board design and code file for the data visualisation interface are made available online (https://github.com/benocd/Temperature-Mapping-Arduino-NTC-Thermistor).

### Fluorescence measurements

Pulse amplitude modulated (PAM) chlorophyll *a* fluorescence measurements were carried out using an Open Fluor CAM imaging system (model: Open FC 800-O/1010-S, Photon Systems Instruments, Brno, Czech Republic). The instrument was programmed to pre-illuminate the sample for 10 minutes with far-red light (730 nm wavelength), followed by 5 minutes of actinic white light and 5 minutes of darkness with saturation pulses applied every minute for determination of F_m_′. Far-red light was switched off during actinic light illumination and switched on again during the subsequent dark period. Actinic light intensity was set at 500 ± 25 μmol photons m^−2^ s^−1^. The complete instrument script for the measurement is available online (Herdean 2021). Yields of regulated (Y(NPQ)) and unregulated (Y(NO)) energy losses were calculated according to Kramer et al. (Kramer et al. 2004).

### Phenoplate measurement

We used the previously described Phenoplate approach (Herdean et al. 2022), with the following modifications: (1) a Gradient Thermal Cycle (Eppendorf 5331 MasterCycler) was used for thermal treatment of the samples; (2) an Open Fluor CAM was used as the imaging system. Briefly, samples from a temperature-controlled room were transferred to the 96-well plate, and the plate was placed on the thermocycler which was set to hold the temperature at the growth temperature of the sample for 10 minutes. During those 10 minutes, the plate was kept in darkness with far-red light on. After the initial 10 minutes, the thermocycler was set to change the temperature to pre-set gradients. Temperature of each column was changed by the thermocycler within 1–3 minutes to the new target values. The PAM fluorometer was configured to start the actinic light and NPQ measurement at the same time as the thermocycler started the temperature change. The temperature gradient was maintained throughout the entire NPQ induction and recovery measurement. To cover the entire temperature range from 10 to 45 °C, we performed measurements on four different temperature gradients with overlapping temperatures Supplementary Fig. 1C.

### Deconvolution and calculation of NPQ components

In this study, we frequently observed values for the first F_m_′ measured in darkness (F_m_′_6_) that were lower than values for the last F_m_′ in light (F_m_′_5_)—a phenomenon that manifests as a positive spike in NPQ upon transitioning to dark. Therefore, we opted to use a dynamic selection of F_m_′ for deconvolution of NPQ components, as previously used to overcome similar issues with other microalgae (Serôdio et al. 2005, 2006), and which we further developed to allow us to better separate different components of NPQ.

Accordingly, the qE-type or fast-relaxing non-photochemical quenching (NPQ) was calculated as the difference between NPQ after 5 minutes of illumination and the minimum NPQ from the subsequent dark adaptation (Müller et al. 2001) according to the following adapted formula:

$$qE\, type\,NPQ\, =\,\left( {\frac{{F_{m} - {F_{m{\kern 1pt}}}^{\prime} _{5}}}{{{F_m{\kern 1pt}}^{\prime } _{5}}}} \right)\,- \,\left( {\frac{F_{m} - max{F_{m{\kern 1pt}}}^{\prime} _{6 - 10}}{max{F_{m{\kern 1pt}}}^{\prime} _{6 - 10}}} \right)$$

where F_m_ is the maximum fluorescence of dark adapted samples after 10 minutes of pre-illumination with far-red light (FR), F_m_*'*_*5*_ is the maximum fluorescence after 5 minutes of illumination with actinic white light, and *maxF*_*m*_*'*_*6–10*_ is the maximum fluorescence recorded from the 5 minutes of dark relaxation after the actinic light illumination. An example measurement is shown in Supplementary Fig. 1A.

The slow-relaxing NPQ was quantified as the minimum NPQ value recorded during dark adaptation after the 5 minutes illumination. The following formula was used:

$$slow \,relaxing\,NPQ\, = \,\left( {\frac{{F_{m} - {maxF_{{m{\kern 1pt}}}}^{\prime } {_{6 - 10{\kern 1pt}}} }}{{{{maxF_{{m{\kern 1pt}}}}^{\prime } _{6 - 10{\kern 1pt}}} }}} \right),$$

The qT_1_-type NPQ was determined as NPQ_(–FR)_-NPQ_(+FR),_ where NPQ_(–FR)_ is the minimum NPQ in the dark relaxation phase in the absence of FR, and NPQ_(+FR)_ is the minimum NPQ in the dark relaxation phase in the presence of FR. The following formula was used:

$$qT_{1} \, type\,NPQ\, = \,\left( {\frac{{F_{m} - {{maxF_{{m{\kern 1pt}}}}^{\prime }{_{ 6 - 10{\kern 1pt}}}} }}{{maxF_{{m{\kern 1pt}}}}^{\prime }{_{ 6 - 10{\kern 1pt}}}} } \right)^{ - FR} {\mkern 1mu} \, - {\mkern 1mu} \,\left( {\frac{{F_{m} - {{maxF_{{m{\kern 1pt}}}}^{\prime }{_{ 6 - 10{\kern 1pt}}}} }}{{maxF_{{m{\kern 1pt}}}}^{\prime }{_{ 6 - 10{\kern 1pt}}}} } \right)^{ + FR}$$

where *–FR* represents values determined in darkness without FR, and + *FR* represents values determined in darkness in the presence of FR.

The qT_2_-type NPQ was determined as the difference between the minimum NPQ value in the dark relaxation phase and the last NPQ value recorded after 5 minutes of dark relaxation. The following formula was used:

$$qT_{2} \,type\,NPQ\,{\mkern 1mu} = \,{\mkern 1mu} \left( {\frac{{F_{m} - {{maxF_{{m{\kern 1pt}}}}^{\prime }{_{ 6 - 10{\kern 1pt}}}} }}{{maxF_{{m{\kern 1pt}}}}^{\prime }{_{ 6 - 10{\kern 1pt}}}} } \right){\mkern 1mu} \, - \,{\mkern 1mu} \left( {\frac{{F_{m} - {{minF_{{m{\kern 1pt}}}}^{\prime }{_{ 6 - 10{\kern 1pt}}}} }}{{minF_{{m{\kern 1pt}}}}^{\prime }{_{ 6 - 10{\kern 1pt}}}} } \right)$$

where *minF*_*m*_*'*_*6–10*_ is the minimum fluorescence recorded in the 5 minutes of dark relaxation after actinic light. Example measurement is shown in Supplementary Fig. 1B.

## Supplementary Information

Below is the link to the electronic supplementary material.

Supplementary file1 (DOCX 2406 KB)

## Data Availability

The datasets generated during and/or analysed during the current study are available from the corresponding author on reasonable request.
