# Supplementary material for: Temperature mapping of non-photochemical quenching in Chlorella vulgaris
Source: Photosynth Res. 2022 Nov 22;155(2):191–202. doi: 10.1007/s11120-022-00981-0 (PMC9879819; doi:10.1007/s11120-022-00981-0)
Supplement: Supplementary file 1 — Supplementary file1 (DOCX 2406 KB) [file 11120_2022_981_MOESM1_ESM.docx]

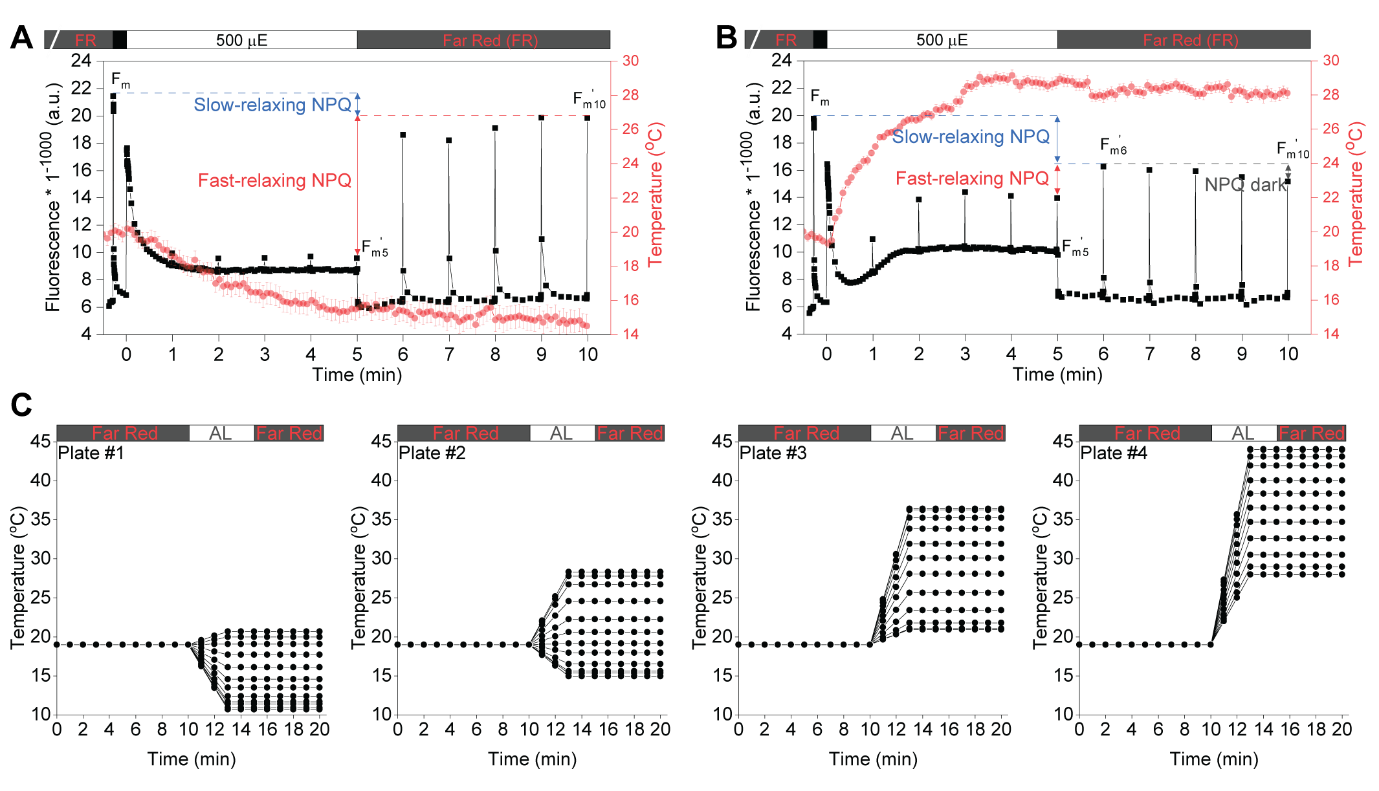


**Supplementary Figure 1. Phenoplate example measurements and NPQ deconvolution strategy.** Example fluorescence traces (black line) from Phenoplate measurement of a 19 ^o^C acclimated *Chlorella vulgaris* sample measured simultaneously in 15.6 ± 0.4 ^o^C (A) and 27.8 ± 0.3 ^o^C (B). Temperature traces (red line) were recorded every 5 s with a FLIR C2 thermal camera and show kinetics of temperature change. Temperature values used in all other figures were extracted from multiple measurements and from the 5 minute time point just before light was turned off. NPQ deconvolution strategy is shown with dashed lines. We assumed that the decrease of *F_m_*′ in the dark relaxation phase under far red light is likely a signature of state transition (qT). We interpret the initial rise of *F_m_*′ at minute 6 as the result of qE relaxation, and State 2 to State 1 transition which is facilitated by the presence of far red light. After minute 6, we observed a quenching of *F_m_*′ which likely represents State 1 to State 2 transition similar to observations seen in *Chlamydomonas reinhardtii* (Allorent et al. 2013). Panel C shows examples of the temperature treatments of the 19 ^o^C acclimated samples.


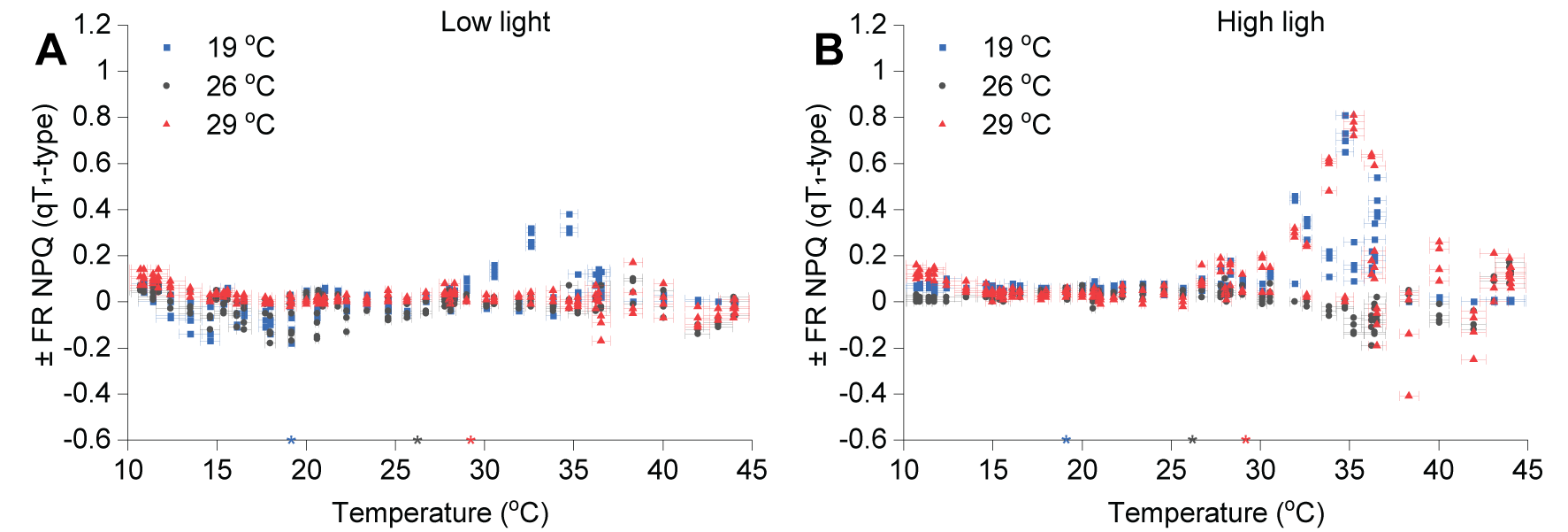


**Supplementary figure 2. qT_1_-type NPQ temperature mapping.** Samples were grown in different temperatures (19, 26 and 29 ^o^C) in low light (A) or high light (B) and measured using the Phenoplate across multiple temperature gradients. Coloured stars on the temperature axis indicate the growth temperature of the three cultures.


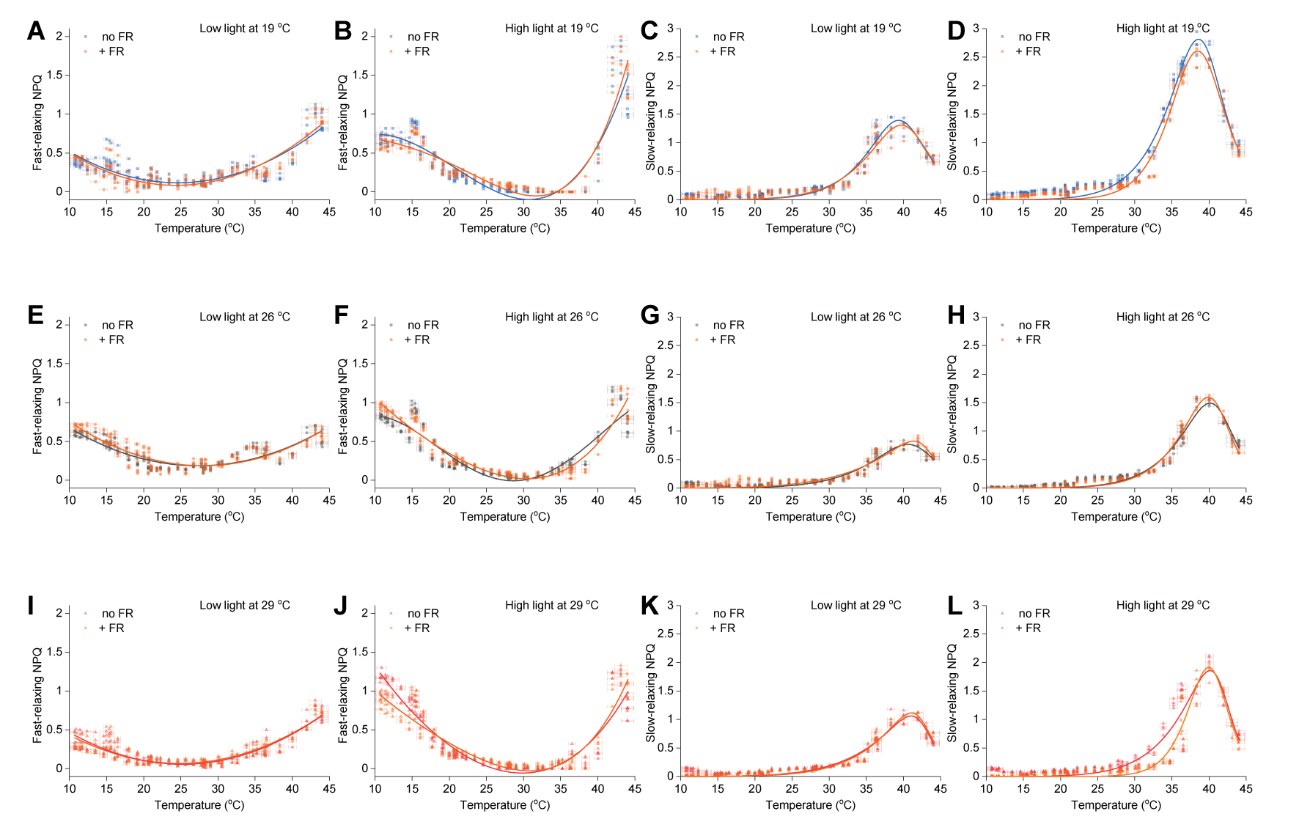


**Supplementary Figure 3. Impact of far red light on fast- and slow-relaxing NPQ.** Samples were grown in different temperatures (19, 26 and 29 ^o^C) in low light or high light and measured using the Phenoplate across a broad temperature gradients. Panels A to D show NPQ from samples acclimated to 19 ^o^C in low- or high-light measured in presence or absence of far red light (FR) in the dark relaxation phase. Panels E to H show data derived from 26 ^o^C acclimated samples, and panels I to L from 29 ^o^C acclimated samples.


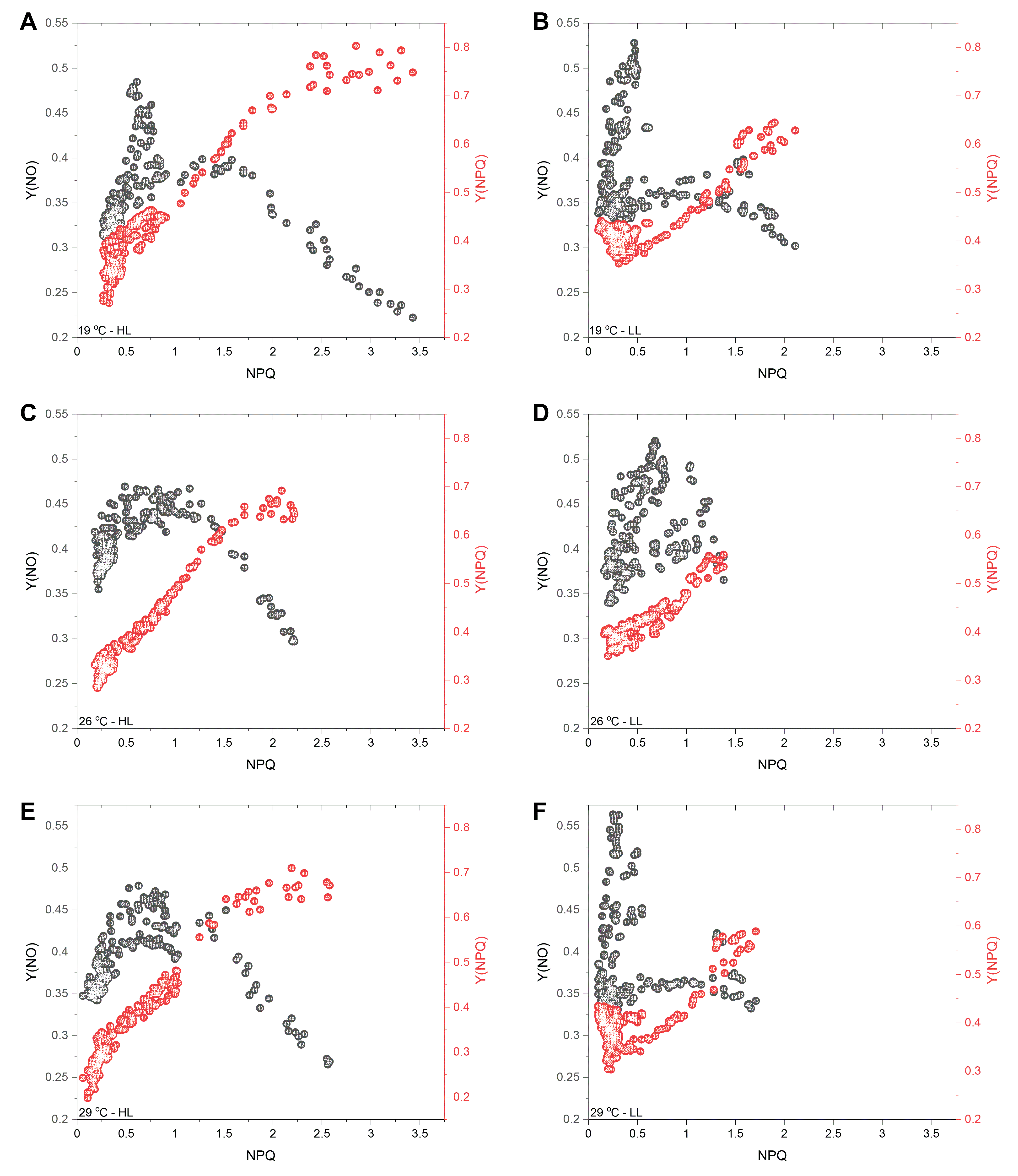


**Supplementary Figure 4. Relationship between NPQ and regulated or unregulated energy dissipation.** Yields of non-photochemical unregulated energy losses (Y(NO)) and photochemical regulated energy quenching (Y(NPQ)) is shown as a function of NPQ. Values were determined after five minutes exposure to high light illumination (500 μmol photons m^-2^ s^-1^) and the various temperatures generated by the Phenoplate. Temperatures for each point is shown in white inside the symbols. Temperature and light acclimation is shown in the bottom left corner of each panel.


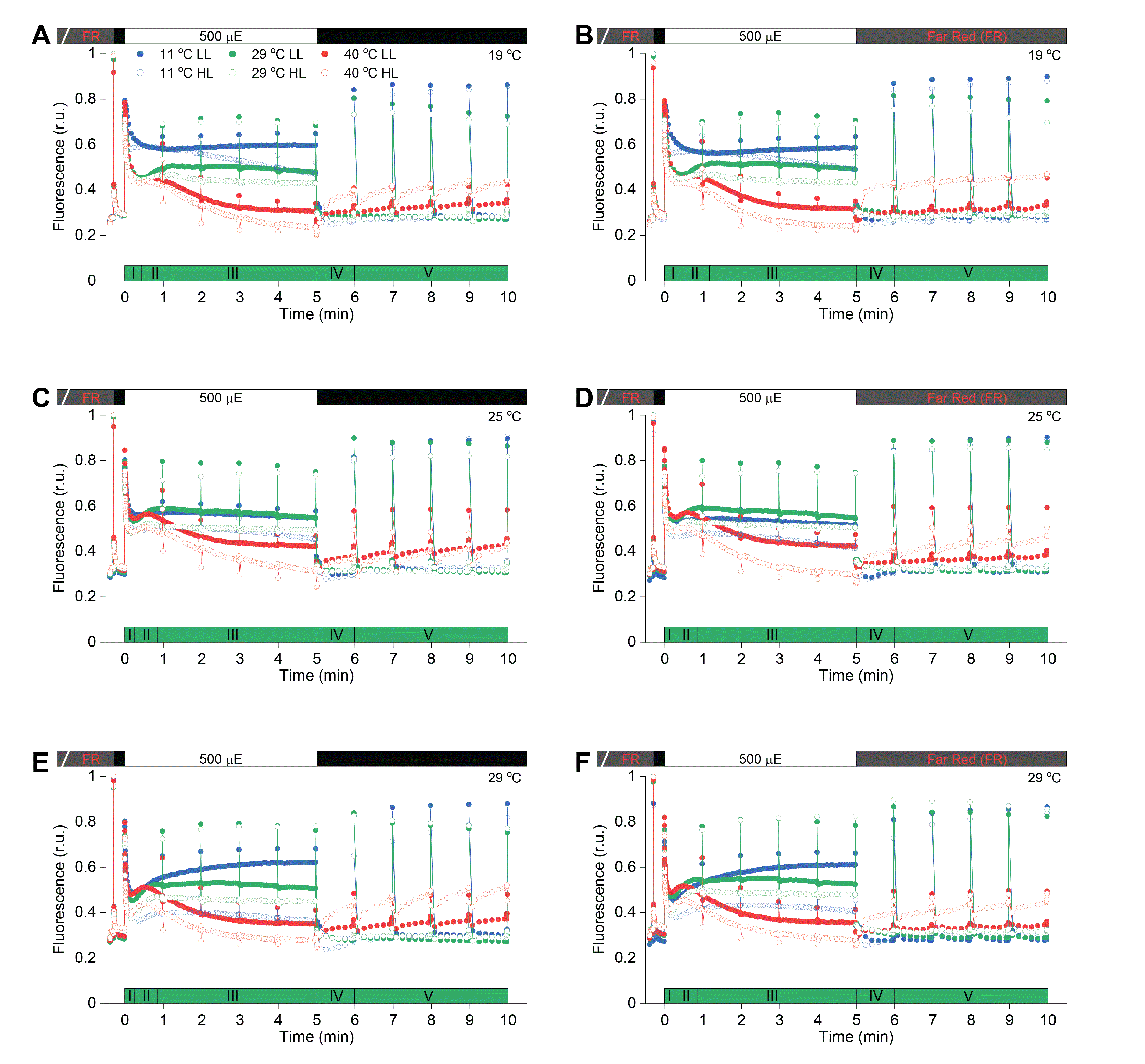


**Supplementary Figure 5. Impact of far red light and temperature on the different phases of fluorescence quenching.** Normalised fluorescence from measurements at 11, 29 and 40 ^o^C in the absence (panels A, C, E) or presence (panels B, D, F) of far red light (FR) in the dark recovery phase. Filled symbols show samples acclimated to low light (LL) and hollow symbols show samples acclimated to high light (HL). Sample acclimation temperature is shown in the top right corner of each panel. Numbered green bars on top of vertical axis show the fluorescence quenching phases of the low light acclimated sample measured at 29 ^o^C. Phase I is described as the fluorescence quenching immediate after onset of illumination, followed by a short recovery known as Phase II. Phase III is described as the subsequent and final fluorescence quenching in the light phase. To resolve the time component of Phases I-III we relied on steady state fluorescence rather than the F_m_′, whereas for Phase IV-V we relied on F_m_′ exclusively.


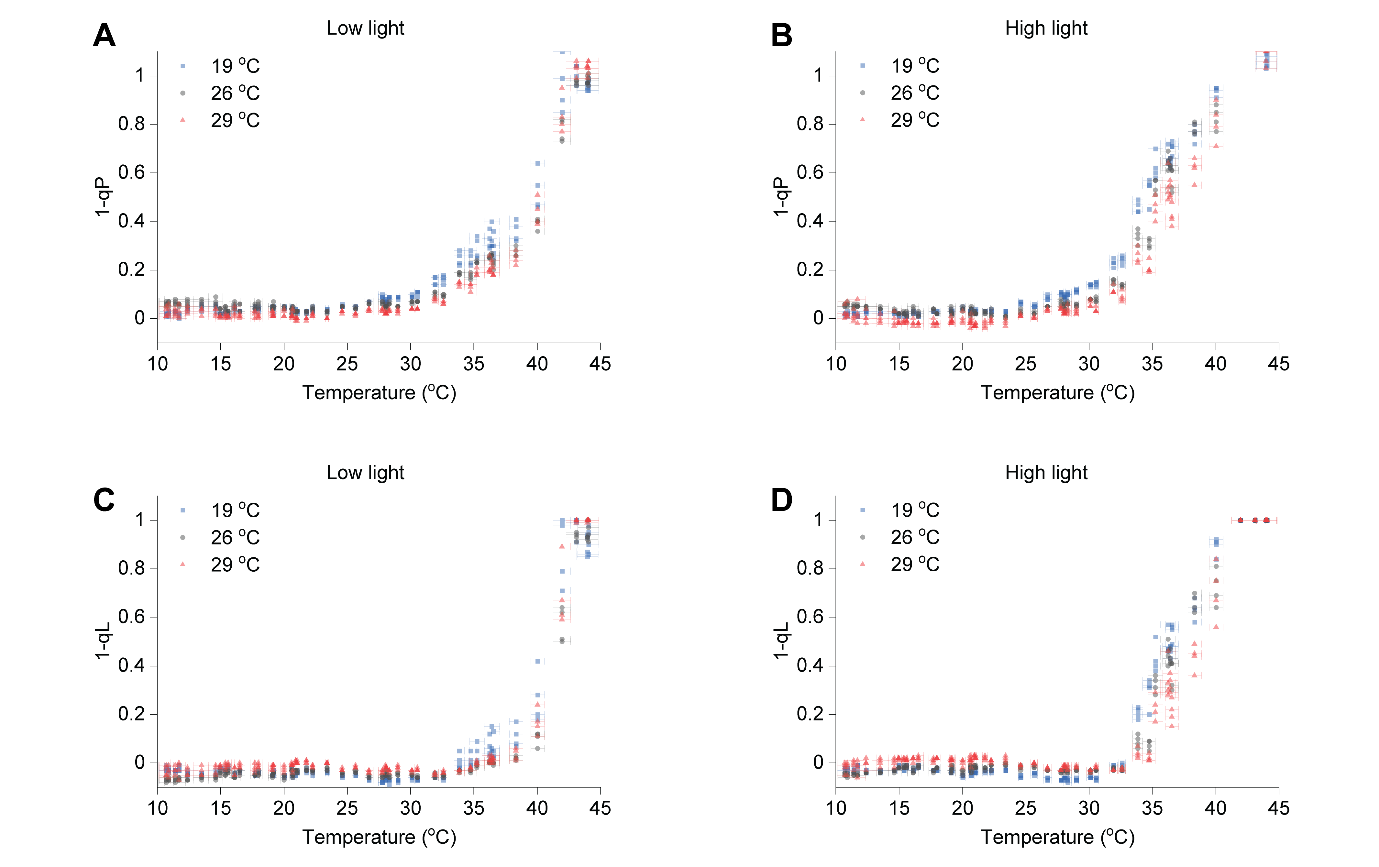


**Supplementary Figure 6. Fraction of open/closed PS II centers.** The fraction of open (0) or closed (1) PSII reaction centers at the end of the 5 minutes dark recovery time point are shown in this figure. Presented values correspond to the same time points shown in Figure 2 for slow-relaxing NPQ. Panels A and B show data calculated using the puddle model for photosynthetic units (1-qP), and panels C and D show parameters calculate using the lake model (1-qL). The acclimation light is shown at the top of each panel (“Low light”; “High light”).


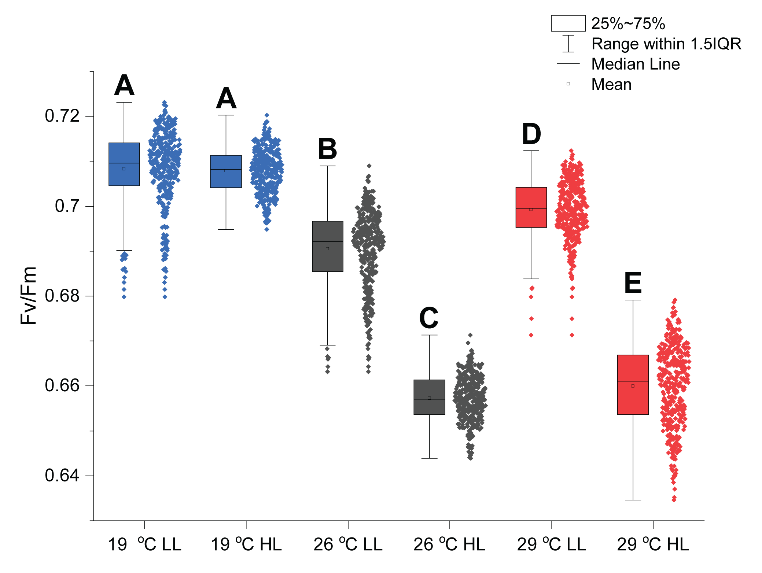


**Supplementary Figure 7. Quantum yield of Photosystem II (Fv/Fm).** Samples were dark adapted for 10 minutes in the presence of far red light and maintained at the acclimation temperature before determination of F_o_ and F_m_. Data points are shown next to the corresponding box plots. Letters on top of box plots indicate statistical differences determined using a One-Way ANOVA analysis with Tukey mean comparison. For each condition we analysed 384 data-points derived from four biological replicates each with 96 technical replicates.

**Supplementary Table 1. Pigment content analysis**

|  | Low light  19 ^o^C | High light  19 ^o^C | Low light  26 ^o^C | High light  26 ^o^C | Low light  29 ^o^C | High light  29 ^o^C |
| --- | --- | --- | --- | --- | --- | --- |
| **Chlorophyll *a:b*** | 2.32 ± 0.19^A,B^ | 2.46 ± 0.17^A^ | 2.11 ± 0.25^A,B^ | 1.49 ± 0.21^A,B^ | 2.40 ± 0.36^A,B^ | 1.35 ± 0.22^B^ |

Samples were collected before the Phenoplate measurement and chlorophyll was extracted according to described protocol. Significant differences between chlorophyll a:b ratios were only observed between high light 19 ^o^C and high light 29 ^o^C samples, with the 29 ^o^C grown sample showing lower values. Letters indicated statistical significant differences determined from ANOVA analysis. Results represent averages from four biological replicates (n=4).
